# Prevalence of Dietary Supplement Use in Healthy Pre-School Chinese Children in Australia and China

**DOI:** 10.3390/nu6020815

**Published:** 2014-02-21

**Authors:** Shu Chen, Colin W. Binns, Bruce Maycock, Yi Liu, Yuexiao Zhang

**Affiliations:** 1 School of Public Health, Curtin University, GPO Box U1907 Perth, Western Australia 6845, Australia; E-Mail: shu.chen2@postgrad.curtin.edu.au; 2 School of Public Health, Curtin University, GPO Box U1907 Perth, Western Australia 6845, Australia; 3 School of Public Health, Curtin University, GPO Box U1907 Perth, Western Australia 6845, Australia; E-Mail: B.Maycock@curtin.edu.au; 4 School of Public Health, Sichuan University, No.17 Section 3 Renmin South Road Chengdu, Chengdu 610041, China; E-Mail: hxliuyi@163.com; 5 Wuhan Maternal and Child Medical Centre, 100 Xianggang Rd, Wuhan 430016, China; E-Mail: zhangyuexiao86@sina.com

**Keywords:** dietary supplements, Chinese, calcium, zinc, migrants, child, nutrition

## Abstract

There is a growing use of dietary supplements in many countries including China. This study aimed to document the prevalence of dietary supplements use and characteristics of Chinese pre-school children using dietary supplements in Australia and China. A survey was carried out in Perth, Western Australia of 237 mothers with children under five years old and 2079 in Chengdu and Wuhan, China. A total of 22.6% and 32.4% of the Chinese children were taking dietary supplements in Australia and China, respectively. In China, the most commonly used dietary supplements were calcium (58.5%) and zinc (40.4%), while in Australia, the most frequently used types were multi-vitamins/minerals (46.2%) and fish oil (42.3%). In Australia, “not working”, “never breastfeed”, “higher education level of the mother” and “older age of the child” were associated with dietary supplement use in children. In China, being unwell and “having higher household income” were significantly related to dietary supplement usage. Because of the unknown effects of many supplements on growth and development and the potential for adverse drug interactions, parents should exercise caution when giving their infants or young children dietary supplements. Wherever possible it is preferable to achieve nutrient intakes from a varied diet rather than from supplements.

## 1. Introduction

Infant nutrition is important for short term and long term health. A balanced variety of nutritious foods are emphasized in the guidelines of the Australian and Chinese governments and other professional organizations as the best source of nutrition for healthy children [1,2,3]. However, the Chinese diet has been reported to be low in calcium, riboflavin, Vitamin A, and zinc [4,5]. A national survey in 2004 found that the average calcium intake among the city and suburban populations was 430 mg per day, well below the recommended intake [6]. The iron intake appears to be adequate in amount, but its bioavailability is very low and consequently the prevalence of iron deficiency and iron deficiency anemia was 43.7% and 7.8%, respectively, among children aged 1–3 years in 2001 [4,7].

The consumption of fortified foods and/or supplements can help some children meet their nutritional needs [8]. Examples of recommended use of supplements include the American Academy of Pediatrics’ recommendation for oral Vitamin D supplementation for exclusively breastfed infants and, under certain conditions, for specific older infants and toddlers [9]. However, other countries, such as Australia, have different climatic conditions and do not recommend universal use of Vitamin D, and the excessive intakes of single nutrients may have the potential for adverse effects [10,11,12].

Dietary supplements enriched with vitamins, minerals, and other substances are increasingly consumed worldwide. The North America and the Asia Pacific regions are the dominant markets for vitamins and dietary supplements [13]. The prevalence of supplement use varies in different ethnic groups for a diversity of dietary and cultural reasons and economic conditions. Most published studies on the use of supplements in children have been conducted in the US and only a small number of studies have been conducted in Asian countries. It is reported that approximately 49% of the U.S. population take dietary supplements and the prevalence of supplement use was 35% among children aged 1–13 years [14,15]. In South Korea, approximately 34% of Korean children and adolescents were taking dietary supplements in a national survey in 2007–2009 [16]. A survey of urban Japanese found that 20.4% of children and adolescents between 3 and 17 years were using supplements, or had used them in the past year [17]. A cross-sectional survey carried out in Zhejiang Province, PR China in 1999 reported a prevalence of 18% of vitamin supplements and 31% of other nutritional supplements in adolescents [18]. A recent study from Taiwan reported that 34.9% of the infants had been given a dietary supplement before six months [19].

Australians have a high prevalence of taking dietary supplements. A representative population survey conducted in 2004 in South Australia reported the use of vitamin supplements by 39.2% respondents and mineral supplements by 13.6% of the population [20]. No recent data is available on the use of supplements by infants or young children in Australia.

Until recently, there have been no reported studies of dietary supplementation among Chinese young children in mainland China or overseas. The aim of this study was to document the prevalence of use of dietary supplements in these populations. A survey was carried out of Chinese mothers living in Perth, Australia and Chengdu and Wuhan, PR China.

## 2. Methods

This data was collected from October 2010 to October 2011 in Perth, Western Australia and from September to December 2011 in Chengdu and Wuhan, China. Participants in Perth were mothers who have at least one pre-school child under 5 years old. They were recruited from the Perth Chinese community, including Chinese schools and community organizations. Mothers interested in taking part in this study received an information sheet containing project details and were asked to sign the consent form. A total of 248 questionnaires were distributed in Perth and 237 mothers agreed to participate (response rate of 95.6%) and 230 mothers completed the dietary supplementation section of the questionnaire. The response rate to the dietary questionnaire was 95.6%. Participants in China were recruited from four kindergartens in four districts of Wuhan and 14 kindergartens in seven districts of Chengdu. Both private and public kindergartens were included. A total of 2400 questionnaires were distributed to mothers by kindergarten teachers and 2079 were returned, a response rate of 86.6%. The dietary supplementation questionnaire was completed by 1464 mothers in China with a response rate to the dietary questionnaire of 70.4%. The study was approved by the Curtin University Human Research Ethics Committee (approval number: HR 96/2010) and the local education authorities in China.

Demographic and dietary supplement use was collected using a validated and reliable questionnaire previously used in Chinese population studies [21]. Pre-coded questions were used to classify income into three groups using categories based on local annual household income surveys [22,23]. A Dietary Supplement Questionnaire is used to collect information on the participants’ use of medicine, vitamins, minerals, herbals, and other supplements during the past two weeks. Detailed information about type, consumption frequency, and amount taken was collected for each reported dietary supplement use. Child’s health status was collected using a translated version of the Australian National Health Survey Questionnaire [24]. 

Body mass index (BMI) was defined as weight (kg)/height (m)^2^. The 2012 revised international child cut-offs developed by the International Obesity Task Force (IOTF) were used to classify thinness, overweightness and obesity in children in this study [25]. They are based on BMI data from six countries, corresponding to the body mass index (BMI) cut-offs at 18 years, which are BMI 25 (overweight), 30 (obesity) and 18.5 (underweight) [25]. 

All statistical analyses were performed using the IBM Statistical Package for Social Sciences (SPSS) Version 20.0. Independent samples’ *t*-test was used to compare means between groups. Mann-Whitney U test was applied to compare the average age of children from two countries. Chi-square (χ^2^) test was used to compare basic characteristics of mothers and children in Australia and China. A multiple binary logistic regression model was used to evaluate the association between mother and child’s characteristics and the use of dietary supplements. A backward elimination procedure was applied to obtain final models. *p* values <0.05 were considered statistically significant.

## 3. Results

A total of 230 Chinese mothers living in Perth, Australia and 1156 mothers living in Chengdu, Sichuan Province and 308 mothers living in Wuhan, Hubei Province, PR China completed the supplements questionnaire. The distribution analysis shows there were no differences between mothers who completed the supplements questionnaire and mothers who did not in age, education attainment, marital status, working status, family income status, breastfeeding initiation and duration. There was also no difference in education attainment, marital status, family income status, breastfeeding initiation and duration, between mothers in Chengdu and Wuhan. The only statistically significant difference between mothers in Wuhan and Chengdu was the average age (31.0 years in Chengdu and 30.8 years in Wuhan, *p* < 0.001). Because the difference is so small in Wuhan and Chengdu mothers, their data were pooled for further analysis.

The average age of Chinese mothers in Australia was older than mothers in China (33.8 ± 4.9 years compared to 31.0 ± 4.1 years, *p* < 0.001). The mothers in Australia also had higher education levels. The median age of the “index child” in the China study population (median age = 3.7 years, the interquartile range = 1.1 years) was older than in Perth (median age = 1.6 years, the interquartile range = 1.9 years, *p* < 0.001). More Perth Chinese children were underweight (22.7%) and fewer overweight and obese (8.0%) than children in China (11.6% underweight and 17.0% overweight and obese, *p* = 0.003) (Table 1).

**Table 1:** Characteristics of Chinese mothers and their children completing dietary questionnaires in Australia and China.

Characteristic	Australia (*n* * = 230)	China (*n* * = 1464)	2-sided *p*-value
	*n* (%)	*n* (%)	
Mothers Age (years)			<0.001
≤30	68 (30.1)	604 (53.3)	
>30	158 (69.9)	530 (46.7)	
Marital status			0.116
Married	229 (99.6)	1151 (98.1)	
Divorced/single/widow	1 (0.4)	22 (1.9)	
Educational attainment			<0.001
High school diploma/TAFE certificate/diploma or less	57 (24.8)	661 (57.1)	
University degree or higher	173 (75.2)	496 (42.9)	
Working status			<0.001
Working	105 (45.7)	968 (83.1)	
Not employed	125 (54.3)	197 (16.9)	
Household income			0.086
Low income	108 (49.5)	572 (55.9)	
High income	110 (50.5)	451 (44.1)	
Mother’s birth place			
Mainland China	187 (81.3)		
Other Asian countries	43 (18.7)		
Duration in Australia (years)			
<5	126 (53.1)		
5–10	73 (32.3)		
>10	33 (14.6)		
Age of the child (years)			<0.001
0–1	62 (27.0)	15 (1.0)	
1–2	81 (35.2)	24 (1.7)	
2–3	38 (16.5)	268 (18.6)	
3–4	30 (13.0)	638 (442)	
4–5	19 (8.3)	497 (34.5)	
Gender of the child			0.737
Boy	122 (53.0)	782 (54.2)	
Girl	108 (47.0)	660 (45.8)	
Weight status of the child (aged 2–4 years old)			0.003
Underweight	20 (22.7)	147 (11.6)	
Normal	61 (69.3)	905 (71.4)	
Overweight/obesity	7 (8.0)	216 (17.0)	
Ever breastfed			
Yes	217 (94.3)	1210 (85.2)	
No	13 (5.7)	211 (14.8)	
Regular exercises			0.002
Yes	117 (60.0)	861 (70.9)	
No	78 (40.0)	353 (29.1)	
Illness during the past 4 weeks			<0.001
Yes	85 (37.3)	790 (55.4)	
No	143 (62.7)	636 (44.6)	
Dietary supplement use by child			0.002
Yes	52 (22.6)	475 (32.4)	
No	178 (77.4)	989 (67.6)	

* The missing values vary for each variable in both countries.

A total of 22.6% of the Chinese children living in Perth were taking dietary supplements, including multi-vitamins/minerals, fish oil, protein, probiotics, colostrum, calcium, zinc and Vitamin AD (or cod liver oil) and Chinese herbs (Table 1). In Chengdu and Wuhan, China, 32.4% of young children were having dietary supplements, including multivitamins/minerals, calcium, zinc, iron, magnesium, fish oil, probiotics, Vitamin A and/or Vitamin D, Chinese herbs or other botanicals (Table 1). Compared to Chinese Australians, Chinese parents living in China were more likely to give their children dietary supplements (χ^2^ = 9.2, df = 1, *p* = 0.002). However, in children aged over 12 months, there is no statistical difference in the prevalence of dietary supplements between Australia (28.6%) and China (32.7%, *p* = 0.284). A higher percentage of children over three years old living in Australia were taking dietary supplements (40.8%) compared to children living in China (31.5%).

In China, the use of calcium supplements was very common among supplement users (58.5%). About half of the Chinese children taking calcium supplements were also taking Vitamin D (*n* = 140, including the use of multi-vitamins). In Australia, only four children were given specific calcium supplements. The most common forms of supplemental calcium used in Chinese children up to five years old are gluconate (51.8%) and carbonate (37.5%). The dosage of calcium supplements ranged from 54–725 mg/day (Table 2). The average intake for calcium carbonate users (307.4 mg/day) is higher than gluconate calcium users (81 mg/day). When calculating the average intake, the intakes from multi-vitamins/minerals were also summed up if they were reported.

**Table 2:** Main dietary supplements used by Chinese children in Australia and China.

Supplement	Australia				China			
	*n*	% supplement users (*n* = 52)	Average intake * (mg/day)	Intake range (mg/day)	*n*	% supplement users (*n* = 475)	Average intake * (mg/day)	Intake range (mg/day)
Calcium	4	9.6	105 (*n* = 5)	75–200	278	58.5	131.4 (*n* = 264)	54–725
Zinc	1	1.9	3.1 (*n* = 12)	1–7.5	192	40.4	4.4 (*n* = 166)	1.62–8.6
Multi-vitamins/minerals	24	46.2	NA	NA	94	19.8	NA	NA
Vitamin A	4	7.7	1026 ** (*n* = 7)	582.5–1617 **	83	17.5	1695 ** (*n* = 71)	600–2800 **
Vitamin D	4	7.7	177 ** (*n* = 5)	85–200 **	91	19.2	568 ** (*n* = 75)	80–780 **
Vitamin C	10	19.2	62.1 (*n* = 12)	20–125	33	6.9	61.4 (*n* = 23)	30–200
Fish oil	22	42.3	859.6 (*n* = 13)	300–1000	4	0.8	NA	NA
Probiotics	2	3.9	NA	NA	22	4.6	NA	NA
Herbs	4	7.7	NA	NA	51	10.7	NA	NA

* When calculated the average intake, the intakes from multi-vitamins/minerals were also summed if they were reported; ** IU/day, IU: international unit; NA: not available.

The prevalence of the use of zinc supplementation was also high in China. Nearly half of supplements users were using zinc supplements (40.4%). Almost all the zinc supplements were in the form of gluconate (93.2%) and the average intake of zinc was 4.4 mg/day (*n* = 166, range from 2.15–8.6 mg) (Table 2).

In Australia, the types most frequently used by supplement users were multi-vitamins/minerals (46.2%) and fish oil (42.3%). The average intake of fish oil was 859.6 mg per day (*n* = 13) with the range from 300 to 1000 mg per day (Table 2).

Chinese herbal supplements were used by children in both countries, especially in China, where 10.7% of supplements users were taking herb supplements (Table 2). Some herbal supplements were used for “better appetite” and some were believed to be beneficial to the immune system or to bring an improvement of health or well-being. In this study, traditional Chinese medicines including cinnabar, as arum, isatis root, kaladana, mangnolia officinalis, scaphium scaphigerum, coltsfoot, coptis chinensis and realgar were included as ingredients in children’s dietary supplements or medicines for (preventing) coughs or colds. Excluding dietary supplements, 7.6% of children in China reported taking medicine during the last two weeks and 82.9% (*n* = 92, 6.3% of all the samples) were taking herbal products for medical reasons, such as cough or upper respiratory tract infection. In China, a total of 16.1% of supplements users (8.6% of the total sample) were using herbal products as dietary supplements or medicine and 7.7% of supplement users (2.2% of the total sample) in Australia reported taking herbal products. 

In 4–5 year old children in Australia, nearly half (47.4%) were taking at least one dietary supplement (Table 3). In Australia, older children (χ^2^ = 19.22, df = 4, *p* = 0.001), children who were never breastfed (χ^2^ = 4.32, df = 1, *p* < 0.05) and children who did regular physical exercises in pre-school or at home (χ^2^ = 10.88, df = 2, *p* = 0.001) were more likely to take dietary supplements than other children. Mothers who had migrated from other Asian regions (including Hong Kong) were more likely to give their children dietary supplements than mothers from mainland China (χ^2^ = 4.47, df = 1, *p* < 0.05) (Table 3).

**Table 3:** Dietary supplement use by children: demographic variables.

	Children used dietary supplements in Australia		Children used dietary supplements in China	
	*n* (%)	2-sided *p*-value	*n* (%)	2-sided *p*-value
Mothers Age (years)		0.201		0.551
<30	12 (17.6)		206 (34.4)	
≥30	40 (25.5)		171 (32.8)	
Education of the mother		0.283		0.942
<University	10 (17.5)		217 (33.1)	
≥University	42 (24.4)		163 (33.3)	
Working status		0.690		0.645
Working	25 (23.8)		321 (33.2)	
Not employed	27 (21.6)		62 (31.5)	
Household income		0.692		<0.001
Low	26 (24.1)		161 (28.1)	
High	24 (21.8)		186 (41.2)	
Mother’s birth place		0.034		
Mainland China	37 (19.9)			
Other Asian regions	15 (34.9)			
Duration in Australia		0.160		
≤5	21 (17.6)			
5–10	21 (28.8)			
>10	9 (27.3)			
Gender of the child		0.868		0.201
Male	28 (23.1)		267 (34.2)	
Female	24 (22.2)		204 (31.1)	
Child’s age (year)		0.001		0.427
<1 year	4 (6.6)		6 (40.0)	
1–2	20 (24.7)		8 (33.3)	
2–3	8 (21.1)		100 (37.5)	
3–4	11 (36.7)		203 (31.8)	
4–5	9 (47.4)		155 (31.2)	
Infant feeding		0.038		0.272
Ever breastfed	46 (21.3)		402 (33.2)	
Never breastfed	6 (46.2)		62 (29.4)	
Child’s BMI		0.406		0.596
Underweight	4 (20.0)		45 (31.7)	
Normal	20 (33.9)		310 (34.3)	
Overweight or obesity	3 (42.9)		64 (31.1)	
Regular exercises		0.001		0.042
Yes	37 (31.6)		306 (35.5)	
No	9 (11.5)		104 (29.5)	
Illness during the past 4 weeks		0.208		0.008
Yes	27 (26.7)		354 (34.8)	
No	25 (19.7)		113 (27.6)	

In China, the prevalence of dietary supplement use was higher in children who had been sick during the past four weeks (χ^2^ = 6.97, df = 1, *p* < 0.01) and children who had regular exercise (χ^2^ = 4.13, df = 1, *p* < 0.05) than in their counterparts. Higher household income was significantly related to the use of child supplements (χ^2^ = 19.29, df = 1, *p* < 0.001) (Table 3).

Mother’s age, education level, working status, household income, the child’s age, BMI, regular exercise, and “illness during the last month” were entered into a binary logistic regression model using backward elimination. After controlling for those potential confounding variables, the results of the binary logistic regression analysis showed that Chinese Australian mothers with higher education levels (OR = 2.51, 95% CI 1.19–5.27), older children (OR = 3.11, 95% CI 1.42–6.83), who were not employed (OR = 3.83, 95% CI 1.09–13.44), and never breastfed their children (OR = 6.75, 95% CI 1.29–35.31) were more likely to give their child dietary supplements. In China, higher household income (OR = 1.53, 95% CI 1.13–2.08) and “having illness during the past month” (OR = 1.44, 95% CI 1.05–1.97) were associated with dietary supplement use in children (Table 4).

**Table 4:** Odds ratios of factors for dietary supplement use in Chinese children in Australia and China.

	China		Australia	
	OR	95% CI	OR	95% CI
Household income			NS	
Low	1			
High	1.53	1.13–2.08		
Education of the mother	NS			
<University			1	
≥University			2.51	1.19–5.27
Working status	NS			
Working			1	
Not employed			3.83	1.09–13.4
Child age (year)			3.11	1.42–6.83
Breastfed	NS			
Yes			1	
Never			6.75	1.29–35.31
Illness during the past 4 weeks				
Yes	1			
No	1.44	1.05–1.97		

NS: not significant.

## 4. Discussion

With the increasing prevalence of chronic disease throughout the world and increasing interest in complementary medicine, dietary supplements have become more widely used in children [26,27]. Many varieties of dietary supplements are now marketed in China and Australia, including single-ingredient products and various combinations of vitamins, minerals, botanicals, and other constituents. Their use in healthy children is addressed towards non-clinical deficiencies, the achievement of optimal status of nutrition and health [17,28].

This study investigated the prevalence of dietary supplement use in Chinese children in mainland China and in Australia. This is the first report, to our knowledge, on the use of dietary supplements in young Chinese children under the age of five years. In this study, one fifth of Chinese children in Perth and one third of children in Chengdu and Wuhan were taking at least one nutritional supplement with no gender differences. The prevalence of dietary supplement use in young children in China was similar to that of the US (35%) and South Korea (34%), but higher than Japan (20.4%) [14,15,16,17]. However, the comparison populations in these reports generally were older children. The lower prevalence of dietary supplement use in Chinese immigrant children in Australia than children in China may be due to the age difference of the subjects. In Australia, most children were under three years old. It was found that older children in Australia were more likely to take dietary supplements.

The types of supplements commonly used in Chinese children in China and in Australia were quite different. In China, calcium and zinc supplements were most commonly used, with many children taking both. Although 58.5% of supplements users were taking calcium supplementation, the average intake was still only 131 mg per day, which is about 20% of the Adequate Intake set for calcium for Chinese children in this age group [3]. It is less than half of the calcium that can be provided from one serve (250 mL) of milk; besides milk can provide other nutrients like protein to support child growth [29]. A meta-analysis on randomized, controlled trials reported little effectiveness of calcium supplementation on bone density in healthy children, either in childhood or later life [30]. The calcium dose was of 300–1200 mg per day in 19 studies included in the meta-analysis, which was much higher than the average calcium intake from supplements in this study (131 mg in China and 105 mg in Australia). Since the level of intake of calcium supplements in China is very low, it is not possible that intake from supplements would be likely to have a positive effect on bone mineral density in Chinese children.

It has been reported in many studies that Chinese children have a low daily zinc intake [31,32]. This may be due to the higher reference value used to define the adequate daily intake in those studies. The Recommended Nutrient Intakes (RNIs) for zinc for 1–7 years old Chinese children range from 9–13.5 mg/day, which are higher than in Japan (5–7 mg/day), USA (3–5 mg/day) and in Australia (3–4 mg/day) [3,29,33,34]. The recommended intake for Chinese children is even higher than the upper level of zinc intakes for those age groups in Australia and New Zealand, which is 7 mg/day for 1–3 years and 12 mg/day for 4–8 years [33]. The 2002 China National Nutrition and Health Survey found that the median intake of zinc in 2–8 year old Chinese children ranged from 5.1 to 7.1 mg/day (the interquartile range: 3.9–9.3 mg/day), which already met the RNIs for this age group in Japan, USA and Australia [35]. However, the adequacy of zinc intake depends not only on the amount, but also its bioavailability. People consuming a diet that provides marginal zinc intake may not absorb an adequate amount of zinc if they are also consuming foods high in phytate together with high calcium [36]. The average population phytate intake of people in China (1186 mg/day) is relatively high compared to their western counterparts, but Chinese diets are low in calcium, reducing the possibility of low zinc availability [35]. The elevation of calcium intake by increasing consumption of milk is not affected by the inhibitory effect of phytate because animal sources of protein appear to promote zinc release from its phytate complex and also provides intrinsic zinc in a highly available form [36]. For young children from this study, their calcium intakes from calcium supplements were low and because of their young age, they still rely on milk products as their main calcium source. Considering the amount of zinc intake from their diet, they may not need to take zinc supplements. Together with the amount of zinc from supplements (ranging from 2.15 to 8.6 mg/day), it is a concern that some children might have reached the upper level of intakes for their age. Adverse events associated with chronic intake of supplemental zinc may include suppression of immune response, decrease in high density lipoprotein cholesterol and reduced copper status [33]. 

In Australia, the most popular supplements were multi-vitamins/minerals, which is consistent with previous studies in children and adolescents [16,17,37]. Fish oil supplements (42.3%) were almost as popular as multi-vitamins and minerals (46.2%). Another large sample size, cross-sectional study (*n* = 266,848) undertaken in New South Wales, Australia also reported a high prevalence of fish oil supplement use in healthy elderly people [38]. Few children were on calcium supplements in Australia. This might be due to higher consumption of milk and milk products in Australia than in China. Commercial advertising may also influence the choice of dietary supplement.

The types of dietary supplements used by young children living in China were distinct from those in Australia. This may be due to the different regulations about supplements that apply to both countries. Promotion and advertising of supplements is different in both countries. There are many reports in the literature that suggest that unnecessary or reckless use of dietary supplements can lead to problems. More studies related to the clinical effectiveness and/or safety of dietary supplements in infants and children are required, especially over the longer term. In the case of Chinese children in China, the intakes of calcium and zinc deserve special considerations in relation to development of dietary supplement regulations. Further studies on fish oil supplements in young children in Australia are also required to add to our knowledge of its health effects.

Herbal products are widely used both in China and by Chinese Australians. Most herbal traditional products not only have plant-derived materials or preparations, but may also include animal products (including scorpions, cicadas and centipedes) and mineral compounds (including cinnabar and realgar) [39]. There is a public perception that these products are inherently safe, however, the therapeutic basis of many ingredients is still not clear. Some traditional ingredients can be toxic when used for inappropriate indications, or prepared inappropriately, or used in excessive dosages, or for a prolonged duration [39,40,41,42]. It is known that some Chinese medicines can have nephrotoxicity or hepatotoxicity effects and some cause increased risk of bleeding [43,44,45,46]. There is a need to increase the awareness of toxic effects of some herbal products in the public and health care professions. 

There are several limitations that need to be considered when interpreting the results of the present study. First, our results may not be representative of all Chinese children in China or in Australia because of the location of the sample and the number of subjects. Secondly, the age distribution of the subjects from two countries in this study was slightly different and this may have a small influence on the results. Nevertheless, we believe our present study to be important for understanding the present status of supplement use in Chinese pre-school children, and in monitoring future trends of supplement use.

## 5. Conclusions

It is important for pre-school children to meet their energy and nutrient needs for growth and development. Consuming a healthy diet is important to achieve adequate nutrient intakes. Dietary supplements only need to be considered when individuals are not able to obtain an adequate nutrient status from their diet alone. A large number of healthy Chinese children both in China and in Australia use dietary supplements, which for most may not be medically indicated. 

Calcium and zinc are the two most popular dietary supplements in young children in China, while multi-vitamin/minerals and fish oil are the most frequently used in Australian Chinese children. However, the supplements used in China contain relatively low amounts of calcium and the same amount could easily be obtained from milk and other dairy products. For some other nutrients such as zinc, the potential over-nutrient of taking supplements should be of concern. There is also a need to increase the awareness of toxic effects of some herbal products in the public and health care professions.

There are many reports in the literature that suggest that unnecessary use of dietary supplements can lead to problems. Parents should exercise caution when giving their infants or young children dietary supplements and be aware of the potential toxicity of inappropriate use or excessive dosages. Before providing dietary supplements, parents should seek advice from appropriate health professionals. For all infants and young children, wherever possible, it is preferable to achieve nutrient intakes from a varied diet rather than from supplements.
